# An Integrated Model of Cardiotoxicity: Interaction of Age, Sex, and Genetic Polymorphisms in Patients with Acute Leukemia

**DOI:** 10.3390/biomedicines14091929

**Published:** 2026-08-28

**Authors:** Muborakkhon Zokirova, Kayumov Abdurakhman, Nargiza Nurillaeva, Jakhongir Khaydarov

**Affiliations:** 1 Republican Specialized Scientific and Practical Medical Center of Hematology, Department of Internal Diseases in Family Medicine №1 and Fundamentals of Preventive Medicine, Tashkent State Medical University, Tashkent 100106, Uzbekistan; 2 Clinical Affairs at the Republican Specialized Scientific and Practical Medical Center of Hematology, Tashkent 100109, Uzbekistan; rahmon555@inbox.ru; 3 Department of Internal Diseases in Family Medicine №1 and Fundamentals of Preventive Medicine, Tashkent State Medical University, Tashkent 100109, Uzbekistan; nargizanur@yandex.ru (N.N.); darkblood0@mail.ru (J.K.)

**Keywords:** acute leukemia, cardiotoxicity, genetic polymorphism, anthracycline, cardiogenetics, cardiovascular complications

## Abstract

**Background:** Cardiotoxic complications represent a major challenge in patients receiving anticancer therapy and may be influenced by interactions among demographic and genetic factors contributing to structural, functional, and electrophysiological myocardial abnormalities. This study aimed to evaluate the effects of age, sex, and genetic polymorphisms on the development of cardiotoxic complications in patients with acute leukemia. **Methods:** A total of 89 patients aged 18–78 years underwent electrocardiography, echocardiography, and molecular genetic analyses of polymorphisms in GPX4, SOD2, COL1A1, CAT, EDN1, and COMT. **Results:** Left ventricular hypertrophy (LVH) was detected in 58.4% of patients, reduced left ventricular ejection fraction (LVEF < 50%) in 23.6%, arrhythmias in 46.1%, and ischemic myocardial changes in 28.1%. Older age was significantly associated with LVH, reduced LVEF, and ischemic myocardial changes (all *p* < 0.05). Sex-specific differences showed a higher prevalence of rhythm disturbances in women and ischemic myocardial changes in men than in women. Individual genetic polymorphisms were significantly associated with specific cardiac phenotypes. Furthermore, combined genetic risk, defined as the presence of two or more unfavorable alleles, was significantly associated with higher rates of LVH, arrhythmias, and impaired myocardial contractility. Multivariate logistic regression identified COMT Val158Met, COL1A1 C1997A, SOD2 C14510A, and EDN1 Lys198Asn as independent genetic predictors of cardiotoxic complications. **Conclusions:** These findings support a polygenic model of cardiotoxicity and highlight the potential value of integrating demographic and genetic factors for personalized cardiovascular risk stratification, early detection, and prevention of treatment-related cardiac complications.

## 1. Introduction

Cardiotoxic complications remain a major cause of adverse outcomes in patients with a wide range of chronic and systemic diseases and in those exposed to pharmacological and metabolic factors. Current evidence indicates that the development of structural and electrophysiological myocardial abnormalities is determined not only by conventional clinical risk factors but also by patient-specific genetic characteristics [1,2,3]. The roles of age and sex as independent modifiers of cardiovascular risk are well established. Advanced age is associated with an increased prevalence of left ventricular hypertrophy, reduced ejection fraction, and ischemic myocardial changes, which are commonly attributed to the cumulative effects of oxidative damage and extracellular matrix remodelling [4,5]. Sex-related differences in cardiac vulnerability are, in turn, largely explained by the influence of sex hormones on neurohumoral regulation, endothelial function, and catecholamine metabolism, as demonstrated in studies by Regitz-Zagrosek et al. (2019) and Mauvais-Jarvis (2020) [6,7].

In recent years, increasing attention has been directed toward genetic polymorphisms that regulate oxidative stress, myocardial fibrosis, and endothelial dysfunction [8]. Polymorphisms in genes encoding antioxidant defense systems, including GPX4, SOD2, and CAT, have been associated with enhanced generation of reactive oxygen species, mitochondrial dysfunction, and an elevated risk of arrhythmias and impaired myocardial contractility, as reported by as reported in previous studies [9,10]. Genes implicated in extracellular matrix remodelling, particularly COL1A1, play a central role in the development of myocardial fibrosis and increased myocardial stiffness. Associations between COL1A1 polymorphisms and left ventricular hypertrophy and reduced ejection fraction have been demonstrated in studies by López et al. (2019) and Mewton et al. (2020) [11,12].

Endothelial dysfunction is also a critical component, mediated in part by polymorphisms in the EDN1 gene, which encodes endothelin-1, a potent vasoconstrictor implicated in the pathogenesis of pulmonary hypertension, myocardial hypertrophy, and ischemic changes. Barton et al. (2020) and Khimji et al. (2022) have shown that unfavorable EDN1 variants are associated with increased vascular tone and impaired coronary perfusion [13,14]. The COMT Val158Met polymorphism has been implicated in altered catecholamine metabolism and neurohumoral regulation, potentially contributing to increased sympathetic activity and cardiovascular dysfunction. These mechanisms may increase susceptibility to tachycardia, arterial hypertension, and cardiac arrhythmia [1,15].

Despite the substantial body of literature, most studies have focused on individual genetic polymorphisms without accounting for their combined effects or the modifying influences of age and sex. At the same time, contemporary concepts of polygenic risk suggest that the cumulative impact of genes involved in antioxidant defense, fibrosis, endothelial function, and neurohumoral regulation determines the severity of structural, functional, and electrophysiological cardiac abnormalities [16,17]. In this context, a comprehensive clinical and genetic analysis appears warranted, incorporating the simultaneous assessment of demographic factors, echocardiographic and electrocardiographic data, and the individual and combined effects of polymorphisms in GPX4, SOD2, COL1A1, CAT, EDN1, and COMT on the development of cardiotoxic complications. We aimed to explore the potential impact of age and sex, as well as the individual and combined effects of selected genetic polymorphisms, on the development of structural and electrophysiological cardiotoxic complications in patients with acute leukemia and to identify candidate risk factors for future validation in larger prospective studies.

## 2. Materials and Methods

The study included patients with the following nosological forms: acute lymphoblastic leukemia (ALL), 52 patients (58.8%); acute myeloid leukemia (AML), 35 patients (39.2%); and acute promyelocytic leukemia (APL), 2 patients (2.0%). The mean age of the patients was 44.8 ± 14.7 years. By gender, the examined were distributed as follows: men, 38 (42.2%); women, 51 (57.8%). Antitumor therapy was used according to the current protocols for treating acute leukemia. The following treatment regimens were most often used: ATRA + ATO, 14 patients; Hyper-CVAD, 9 patients; 7 + 3 protocol, 8 patients; high-dose cytarabine (HiDAC), 13 patients; azacitidine-containing regimens, 13 patients; and other treatment regimens, 32 patients.

For the purposes of this study, the primary composite cardiovascular endpoint comprised clinically relevant structural, functional, electrophysiological, and biochemical cardiac abnormalities identified during the observation period of the study. These include reduced left ventricular ejection fraction (LVEF), left ventricular hypertrophy (LVH), clinically significant rhythm and conduction disorders, ischemic electrocardiographic abnormalities, elevated cardiac troponin levels, increased NT-proBNP concentrations, and echocardiographic evidence of myocardial remodelling.

According to the 2022 ESC Cardio-Oncology Guidelines, reduced LVEF, elevated cardiac biomarkers, and newly developed myocardial dysfunction are the principal manifestations of cancer therapy-related cardiac dysfunction (CTRCD). However, the present study aimed to evaluate the overall cardiovascular phenotype and genetic susceptibility to cardiotoxic complications in patients with acute leukemia. Therefore, structural abnormalities, such as LVH and ischemic ECG changes, were retained in the composite endpoint as markers of cardiovascular vulnerability that may influence the development and clinical expression of treatment-related cardiotoxicity, rather than as specific indicators of therapy-induced myocardial injury.

The control group consisted of 97 healthy volunteers with no history of oncohematological diseases, chronic cardiovascular pathology, autoimmune diseases, or severe somatic disorders. The control group comprised healthy blood donors, medical institution employees, and individuals who underwent preventive medical examinations and belonged to the same ethnic population as the patients in the study group. The inclusion criteria for the control group were as follows: age > 18 years, no history of malignant neoplasms, no blood system diseases, and no clinically significant cardiovascular pathology. The exclusion criteria were as follows: any cancer, blood system diseases, chronic heart failure, coronary heart disease, congenital heart defects, severe chronic liver and kidney diseases, and systemic inflammatory diseases. Comparability of the groups: To minimize bias, the control group was selected for demographic comparability. Comparative analysis showed no statistically significant differences between the study and control groups in terms of sex or age. The differences in sex and age between the groups were not statistically significant (*p* > 0.05). Ethical aspects: The study was conducted in accordance with the principles of the Declaration of Helsinki of the World Medical Association. The study protocol was approved by the local bioethics committee of the Republican Specialized Scientific and Practical Medical Center for Hematology. All study participants provided voluntary informed consent to participate and undergo molecular genetic analysis before being included in the study.

The exclusion criteria were congenital heart defects, acute inflammatory myocardial diseases, and absence of key clinical or genetic data. Electrocardiographic Assessment: Before initiating chemotherapy, all patients underwent a baseline cardiovascular assessment, including a standard 12-lead electrocardiogram (ECG), transthoracic echocardiography, and measurement of cardiac troponin and NT-proBNP. No patient had clinically significant cardiovascular abnormalities that required modification or postponement of induction chemotherapy according to institutional treatment protocols. The baseline assessment served as a reference for the subsequent evaluation of cardiovascular changes during treatment.

Follow-up cardiovascular assessment using the same investigations was performed after the completion of induction chemotherapy, which is the most intensive and potentially cardiotoxic phase of treatment for acute leukemia, aimed at achieving complete remission and reducing the bone marrow blast burden. Cardiovascular abnormalities detected after induction therapy.

Standard 12-lead ECG recordings were obtained at rest and were subsequently interpreted by a cardiologist. The following parameters were analyzed: sinus tachycardia, ventricular and supraventricular premature beats, rhythm and conduction disturbances, ischemic changes in the ST segment and T wave, signs of post-infarction cardiosclerosis, and low-voltage QRS complexes. Arrhythmias were classified according to the generally accepted clinical criteria.

Echocardiographic Assessment [18]: Transthoracic echocardiography (TT EchoCG) was performed using M-mode, B-mode, and Doppler imaging in accordance with a standard protocol. The following parameters were evaluated: interventricular septal thickness (IVST), left ventricular posterior wall thickness (LVPWT), left ventricular myocardial mass (LVMM), left ventricular end-diastolic and end-systolic dimensions and volumes, left ventricular ejection fraction (LVEF), dimensions of the left atrium and aorta, presence of segmental hypokinesia, signs of left ventricular hypertrophy (LVH), pulmonary hypertension, and pericardial effusion. Reduced LVEF was defined as a value of <50%. Molecular Genetic Analysis: Peripheral venous blood samples were used for molecular genetic analysis. Genomic DNA was extracted using standard protocols.

The studied polymorphisms were divided into functional groups: antioxidant protection SOD2 C14510A, GPX4 C718T, and CAT G262A. These genes are involved in neutralizing reactive oxygen species and protecting cardiomyocytes from oxidative damage. Endothelial Dysfunction and Myocardial Remodelling: EDN1 Lys198Asn and COL1A1 C1997A—These genes are involved in vascular remodelling, myocardial fibrosis, and endothelial dysfunction. Neurohumoral regulation: COMT Val158Met—The COMT polymorphism affects catecholamine metabolism and can determine an individual’s cardiovascular system sensitivity to stress and anticancer therapy. Peripheral venous blood was used for the genetic analysis. Blood samples were collected in EDTA-containing vacuum tubes. Genomic DNA was isolated from the leukocyte fraction of the blood using a commercial DNA extraction kit according to the manufacturer’s instructions. The concentration and purity of the isolated DNA were determined using spectrophotometry.

Genotyping of the studied polymorphisms was performed using real-time polymerase chain reaction (PCR) with allele-specific TaqMan probes. Amplification was performed according to the manufacturer’s instructions. The following quality control procedures were used to ensure the reliability of the results: inclusion of negative controls in each PCR batch, repeated genotyping of 10% of randomly selected samples, independent verification of the results by two investigators, and automated quality control of amplification curves. The genotyping reproducibility rate was >99%. Genotype determination was successful in more than 98% of the examined individuals, and the frequency of missing genotypes for each polymorphism did not exceed 2%. Missing data did not significantly affect the analyses. None of the polymorphisms studied showed a deviation from the Hardy–Weinberg equilibrium (*p* > 0.05).

To reduce the probability of first-type errors, the False Discovery Rate (FDR) procedure based on the Benjamini-Hochberg method was also used. After correction, the main statistically significant associations remained significant. The results were most stable for the following (Table 1):

The results confirmed the stability of the identified genetic associations. Combined genetic risk was defined as the presence of two or more adverse alleles among the studied polymorphisms. This approach is based on the concept of cumulative genetic burden, which is widely used in cardiovascular genetics and pharmacogenetics. The use of an integral indicator allows for consideration of the polygenic nature of cardiovascular complications and better reflects the biological mechanisms of cardiotoxicity than the analysis of individual genes.

To prevent overfitting, the number of variables included in the multivariate analysis was limited according to the events per variable (EPV) principle. In the study group, 64 cases of cardiovascular complications were recorded. Given the number of events, only variables that demonstrated statistical significance in the univariate analysis were included in the final model. The final model included age, NT-proBNP, troponin, COMT Val158Met, COL1A1 C1997A, SOD2 C14510A, and EDN1 Lys198Asn. This ensured the statistical stability of the model and avoided overfitting. As part of the preparation of the revised version of the manuscript, the following were re-checked: correlation coefficients, *p*-values, confidence intervals, ROC analysis indicators, and results of logistic regression. All statistical calculations were double-checked and presented in a single format. The results are presented in Table 2.

### Statistical Analyses

Continuous variables are reported as mean ± standard deviation or median with interquartile range, as appropriate. Categorical variables are presented as frequencies and percentages. Differences between groups were assessed using appropriate statistical tests for continuous and categorical variables. The associations between demographic characteristics, including age and sex, and individual cardiac phenotypes were evaluated.

The frequencies of genetic polymorphisms and alleles were analyzed in patients and controls. Genotype distributions were tested for conformity with Hardy–Weinberg equilibrium, and allele and genotype frequencies were compared between the groups. Associations between individual genetic variants and cardiac complications were evaluated, and odds ratios (ORs) and 95% confidence intervals (CIs) were calculated where appropriate.

Multivariable logistic regression analysis was performed to determine the independent associations between genetic polymorphisms and cardiotoxic complications. The results of the regression models were expressed as regression coefficients (β), odds ratios (ORs), 95% confidence intervals (CIs), and *p* values. The performance of the multivariable model was assessed using the model chi-square statistic, Nagelkerke’s R^2^, receiver operating characteristic (ROC) curve analysis, area under the ROC curve (AUC), sensitivity and specificity. All statistical tests were two-sided, and a *p*-value < 0.05 was considered statistically significant.

In the present study, cardiotoxicity was defined according to the recommendations of the European Society of Cardiology (ESC Cardio-Oncology Guidelines, 2022) [19], which was considered to indicate the development of structural, functional, electrophysiological, or biochemical signs of cardiovascular injury in patients with cancer receiving antitumor therapy. A combined endpoint was used to assess cardiotoxicity, including: reduction in LV ejection fraction; myocardial remodelling; clinically significant arrhythmias; increase in troponin; increase in NT-proBNP [20]; and echocardiographic signs of myocardial dysfunction that were recorded.

## 3. Results

A total of 89 patients aged 18–78 years were included, with a median age of 52 years (IQR 36–63). The study cohort constituted a pragmatic clinical population of patients with cardiac abnormalities in whom structural myocardial changes (assessed by echocardiography) coexisted with electrical abnormalities (assessed by ECG).

A high prevalence of left ventricular hypertrophy (LVH, 58%) indicates the predominant role of myocardial remodelling, likely associated with chronic hemodynamic overload, endothelial dysfunction, and fibrosis. Reduced left ventricular ejection fraction (LVEF, 24%) suggests a substantial proportion of patients with systolic dysfunction, reflecting an increased risk of heart failure progression. Arrhythmias (46%), including tachycardia, ventricular premature beats, and supraventricular ectopy, demonstrate that impaired electrical stability of the myocardium is one of the most common manifestations of cardiotoxicity. Ischemic changes/post-infarction cardiosclerosis (28%) confirm that a significant proportion of patients exhibit either ischemic mechanisms of myocardial injury or post-ischemic remodelling. The demographic and clinical characteristics of the study population are presented in Table 3.

Age. The observed correlations exhibited a consistent and biologically plausible trend: with increasing age, the prevalence of LVH increased (*p* = 0.001), LVEF declined (*p* = 0.003), and ischemic changes were more frequently detected (*p* = 0.006). Age appears to be a strong independent factor reflecting cumulative myocardial damage, increased oxidative stress, fibrosis progression, and vascular dysfunction.

Sex. Sex-related differences were identified as follows: women more frequently exhibited rhythm disturbances (*p* = 0.041), whereas men more often demonstrated ischemic changes/post-infarction cardiosclerosis (*p* = 0.048). Thus, sex acts as a modifier of the clinical phenotype: electrical instability (tachyarrhythmias, ectopic beats) predominates in women, whereas an ischemic pattern of myocardial injury is more typical in men. These data are consistent with a model in which neurohumoral and autonomic mechanisms prevail in women, whereas coronary and structural components predominate in men.

Women comprised approximately 60% of the study population. According to echoCG and ECG data, LVH was detected in 58% of patients; reduced LVEF (<50%) in 24%; rhythm disturbances (ventricular/supraventricular ectopy, tachycardia) in 46%; and ischemic changes/post-infarction cardiosclerosis in 28%.

Correlation analysis demonstrated a positive correlation between age and LVH (r = 0.41; *p* = 0.001); a negative correlation between age and LVEF (r = 0.36; *p* = 0.003); and an association between age and ischemic changes (r = 0.33; *p* = 0.006).

Women were significantly more likely to have rhythm disturbances (52.8% vs. 36.1%; *p* = 0.041) while men more frequently exhibited ischemic changes/post-infarction cardiosclerosis (36.1% vs. 22.6%; *p* = 0.048). The associations between age, sex, and cardiac complications are presented in Table 4.

Associations of Individual Genetic Polymorphisms Antioxidant Defence Genes

— GPX4 (T allele) was associated with reduced LVEF (OR 2.3; *p* = 0.021);
— SOD2 (A allele) with arrhythmias (OR 2.1; *p* = 0.028);
— CAT (A allele) with ischemic changes (OR 2.0; *p* = 0.037).

Genes of Remodelling and Regulation

— COL1A1 (A allele) was associated with LVH (OR 2.6; *p* = 0.009);
— EDN1 (Asn allele) with pulmonary hypertension and tachycardia (*p* = 0.031);
— COMT (Met/Met genotype) with tachycardia and arrhythmias (OR 2.4; *p* = 0.015).

The combined allelic and genotypic distributions of the studied genetic polymorphisms are presented in Table 5.

The results of the multivariable logistic regression analysis are presented in Table 6.

The performance of the multivariable logistic regression model is presented in Table 7.

Multivariable logistic regression identified the COMT Met, COL1A1 A, EDN1 Asn, and SOD2 A alleles as independent genetic predictors of cardiotoxic complications. The strongest association was observed for COMT Val158Met (OR = 3.83, *p* = 0.001).

To determine the independent contribution of the studied genetic factors to the development of cardiovascular complications in patients with acute leukemia, multivariate logistic regression analysis was performed. The model included polymorphisms in antioxidant system genes (SOD2, GPX4, and CAT), endothelial dysfunction and myocardial remodelling (EDN1 and COL1A1), and the gene for neurohumoral regulation, COMT.

The analysis showed that the COMT Val158Met polymorphism made the greatest contribution to the development of cardiovascular complications in patients with schizophrenia. Carriage of the Met allele was associated with a 3.83-fold increase in the risk of cardiovascular complications (OR 3.83; 95% CI 2.11–6.95; *p* = 0.001), indicating a significant role of neurohumoral dysfunction in the pathogenesis of cardiotoxicity in patients with acute leukemia.

The second most important independent predictor was the COL1A1 C1997A variant. The presence of a risk allele was associated with a 2.53-fold increase in the likelihood of cardiovascular complications (OR 2.53; 95% CI 1.60–4.00; *p* = 0.001). The data obtained confirm the important role of myocardial remodelling and fibrosis in the development of cardiac dysfunction.

A statistically significant association was also identified for the SOD2 polymorphism, C14510A. Carriage of the A-allele increased the chance of complications by 1.9-fold (OR = 1.90; 95% CI 1.21–2.95; *p* = 0.010), indicating the importance of mitochondrial oxidative stress in the mechanism of cardiac damage.

The EDN1 polymorphism Lys198Asn also retained independent prognostic significance after adjusting for other genetic factors. The presence of an unfavorable variant was associated with a 1.78-fold increase in the risk of cardiovascular complications (OR = 1.78; 95% CI 1.05–3.01; *p* = 0.030), indicating the involvement of endothelial dysfunction and vascular remodelling in the development of cardiovascular disorders.

For GPX4 C718T polymorphism, there was a trend towards an increased risk of cardiovascular complications (OR = 1.47; 95% CI 0.94–2.30), but statistical significance was not achieved (*p* = 0.089). Similarly, the CAT G262A polymorphism did not show an independent effect on the chance of complications (OR = 1.05; 95% CI 0.58–1.89; *p* = 0.590).

The quality assessment of the model showed a high predictive ability. The overall statistical significance of the model was $\chi^{2}$ = 31.8, *p* < 0.001. The Nagelkerke coefficient of determination was 0.42, indicating that approximately 42% of the variation in the risk of cardiovascular complications was explained by the genetic factors included in the model.

The model’s diagnostic performance was characterized by an area under the ROC curve (AUC) of 0.85, a sensitivity of 76.3%, and a specificity of 78.6%. The data show a strong prognostic value of the complex genetic model and confirm the potential of the studied genetic markers for the early prediction of cardiovascular complications in patients with acute leukemia.

Thus, the most significant independent genetic predictors of cardiovascular complications in patients with acute leukemia were COMT Val158Met, COL1A1 C1997A, SOD2 C14510A, and EDN1 Lys198Asn polymorphisms. The greatest contribution to risk formation was made by the COMT Val158Met polymorphism, which emphasizes the important role of neurohumoral dysfunction in the pathogenesis of cardiotoxicity. The associations between individual genetic polymorphisms and cardiac complications are presented in Table 8.

Combined Genetic Effects (Main Finding). Patients were stratified into two groups:

— 0–1 unfavourable allele;
— ≥2 unfavourable alleles.
  Key findings:
— LVH: 72.5% vs. 38.9% (*p* < 0.001);
— Arrhythmias: 61.3% vs. 29.6% (*p* = 0.002);
— Reduced LVEF: 35.4% vs. 12.9% (*p* = 0.008).

Multivariable Analysis. Logistic regression analysis identified the following independent predictors of cardiotoxic complications: age (*p* = 0.002); combined genetic risk (*p* < 0.001); and sex (for arrhythmias, *p* = 0.044).

## 4. Discussion

The present study shows that cardiotoxic complications are multifactorial and determined not only by age and sex but also by genetic predisposition, particularly in the presence of an unfavorable combined genetic profile. These findings support the concept of polygenic risk, according to which clinically significant cardiovascular abnormalities are more likely to develop from the cumulative effects of multiple gene variants involved in antioxidant defense, myocardial remodelling, endothelial function, and neurohumoral regulation.

The methodological limitations of the present study should be acknowledged. Certain components of the composite endpoint, particularly left ventricular hypertrophy and ischemic electrocardiographic abnormalities, may reflect pre-existing cardiovascular disease rather than the direct manifestations of cancer therapy-related cardiac dysfunction. Nevertheless, these variables were intentionally included because the study aimed to characterize the overall cardiovascular phenotype and investigate whether genetic polymorphisms modify susceptibility to clinically relevant cardiovascular abnormalities during anticancer treatment. Consequently, these findings should be interpreted as indicators of cardiovascular vulnerability rather than as exclusive markers of treatment-induced myocardial injury.

Age and Sex as Modifiers of Cardiotoxicity Risk [21]. In our study, age was significantly associated with LVH, reduced LVEF, and a higher frequency of ischemic changes. This pattern can be explained by age-related increases in oxidative stress, progression of vascular dysfunction, and myocardial fibrosis. In the context of cardiotoxicity, age may act as a “background amplifier” of injury, against which genetic factors exert a more pronounced effect.

Sex-related differences were also clinically relevant: rhythm disturbances were more frequent in women, whereas ischemic changes and post-infarction cardiosclerosis were predominant in men. This phenotypic shift indicates that sex influences not only the incidence but also the pattern of cardiac complications (electrical vs. ischemic), a distinction that should be considered when developing monitoring strategies.

Antioxidant defense genes (GPX4, SOD2, CAT). One of the central mechanisms of cardiotoxic myocardial injury is an imbalance between reactive oxygen species production and antioxidant defense [22]. In this study, polymorphisms in antioxidant genes were associated with a higher incidence of arrhythmias, impaired contractile function, and ischemic changes.

Particular attention should be paid to GPX4, which plays a key role in protecting the cellular membranes from lipid peroxidation. Recent studies have recognized GPX4 as a potential modifier of cardiovascular risk; variants in this gene have been linked to altered enzyme activity and an increased likelihood of postoperative atrial fibrillation. These results support the role of GPX4 as a marker of myocardial vulnerability under oxidative stress conditions.

Regarding SOD2, recent reviews on the genetic determinants of cardiotoxicity have emphasized the importance of mitochondrial oxidative stress as a trigger of arrhythmogenesis and cardiomyocyte dysfunction. This is consistent with our data, which show that unfavorable SOD2 variants are associated with electrical instability of the myocardium, including ventricular and supraventricular ectopy.

The CAT gene is responsible for hydrogen peroxide detoxification and contributes to vascular homeostasis. Reduced antioxidant capacity increases the likelihood of endothelial injury and impairs coronary perfusion, which may manifest clinically as ischemic ECG changes. Our findings further support the hypothesis that antioxidant polymorphisms are relevant not only to arrhythmias but also to the ischemic component of cardiotoxicity.

COL1A1 and Myocardial Remodelling: Association with Fibrosis and LVH Hypertrophy. The strong association between COL1A1 polymorphism and LVH observed in our cohort illustrates the role of genetically determined alterations in extracellular matrix remodelling. COL1A1 encodes type I collagen, which is a key structural component of the myocardial interstitium. Increased fibrotic potential contributes to myocardial stiffness, impaired diastolic function, and hypertrophy progression.

Recent studies have emphasized the role of profibrotic phenotypes of cardiac fibroblasts and key genes associated with fibrosis progression in ischemic heart disease. This supports the relevance of including fibrosis-related genes, such as COL1A1, in risk evaluation models for structural cardiotoxicity and underscores the potential to develop genetic panels for early risk stratification.

EDN1 as a Marker of Endothelial Dysfunction and Hemodynamic Load. In our study, the EDN1 polymorphism was associated with signs of pulmonary hypertension and tachycardia in the patients. Endothelin-1 is one of the most potent endogenous vasoconstrictors and plays a central role in regulating vascular tone, vascular remodelling, and myocardial hypertrophy. Genetic variants of EDN1 may alter vascular reactivity, thereby contributing to the development of hypertrophy and secondary hemodynamic overload. From a clinical perspective, this is particularly relevant because EDN1 links molecular mechanisms to echocardiographic phenotypes, including elevated pulmonary pressure, hypertrophy, and compensatory tachycardia.

COMT Val158Met: Neurohumoral Regulation and Electrical Instability. Our findings show an association between the COMT Val158Met polymorphism and rhythm disturbances and tachycardia, consistent with the pathogenic concept of increased sympathetic activation in the setting of reduced COMT activity and impaired catecholamine metabolism. Previous studies have shown that the COMT Val158Met polymorphism may be associated with age-dependent changes in parasympathetic control. This is particularly relevant for interpreting our results, as the electrical instability of the heart in clinical practice frequently results from the combined influence of age, stress, and neurohumoral activation. Accordingly, COMT may be considered a factor that increases the arrhythmogenic phenotype when other unfavorable genetic variants are present.

Combined Genetic Effects as Key Predictors of Phenotypic Severity. The principal finding of this study was that combined genetic risk (carriage of $\chi^{2}$ 2 unfavorable alleles) was associated with a higher prevalence of LVH, arrhythmias, and reduced LVEF, with the effect remaining significant after adjustment for age and sex. This observation is of fundamental importance as it indicates that clinically significant cardiotoxicity more often develops through the convergence of multiple pathogenic pathways.

— Antioxidant deficiency (GPX4/SOD2/CAT) → increased oxidative stress.
— Fibrosis and remodeling (COL1A1) → myocardial stiffness and hypertrophy.
— Endothelial dysfunction (EDN1) → vasoconstriction and hemodynamic overload.
— Neurohumoral hyperactivation (COMT) → tachycardia and electrical instability.

This integrative framework is consistent with current research trends in the field of genetic determinants of cardiotoxicity, where the importance of multiple interacting genetic factors is increasingly recognized.

The results suggest that combined polymorphisms in GPX4, SOD2, COL1A1, CAT, EDN1, and COMT may serve as a basis for identifying high-risk patients, implementing intensified ECG and echocardiographic monitoring, and initiating early prophylactic strategies to limit progressive remodelling and arrhythmias.

The application of a clinical-genetic model appears especially promising in cases where cardiotoxicity may develop rapidly or remain subclinical, thereby requiring a personalized approach to risk assessment and monitoring.

Our findings support the growing concept that integrating genetic information with clinical and imaging parameters may improve individualized cardiotoxicity risk assessment, complementing the current cardio-oncology surveillance strategies proposed in recent expert reviews [23].

Future studies should aim to validate the biological significance of the identified genetic variants using advanced experimental models. In particular, induced pluripotent stem cell-derived cardiomyocytes (iPSC-CMs) and three-dimensional cardiac organoids may provide valuable platforms for investigating the influence of high-risk genetic polymorphisms on oxidative stress, myocardial remodelling, endothelial dysfunction, and cellular responses to anthracycline exposure. Such mechanistic studies would complement the present clinical findings and advance our understanding of the molecular pathways underlying individual susceptibility to cardiotoxicity.

## 5. Conclusions

**Figure 1:**
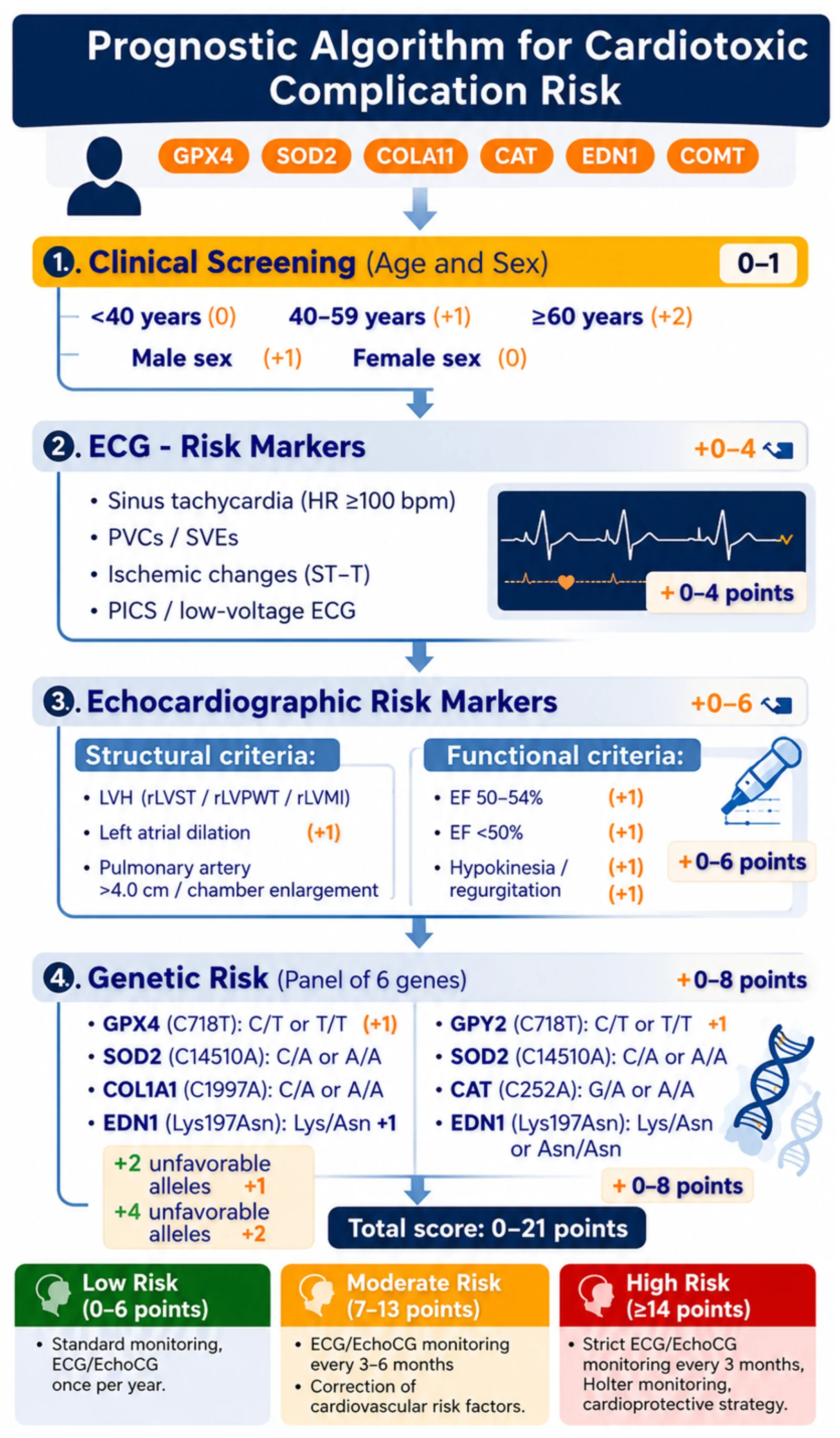
Figure prognostic algorithm for cardiotoxic complication risk stratification based on clinical, electrocardiographic, echocardiographic, and genetic parameters. The figure uses color coding to distinguish the clinical, electrocardiographic, echocardiographic, and genetic risk-assessment components and the final low-, moderate-, and high-risk categories.

The findings of this exploratory study suggest that age, sex, and selected genetic polymorphisms may contribute to individual susceptibility to cardiotoxicity-associated cardiovascular abnormalities in patients with acute leukemia (Figure 1).

**Figure 2:**
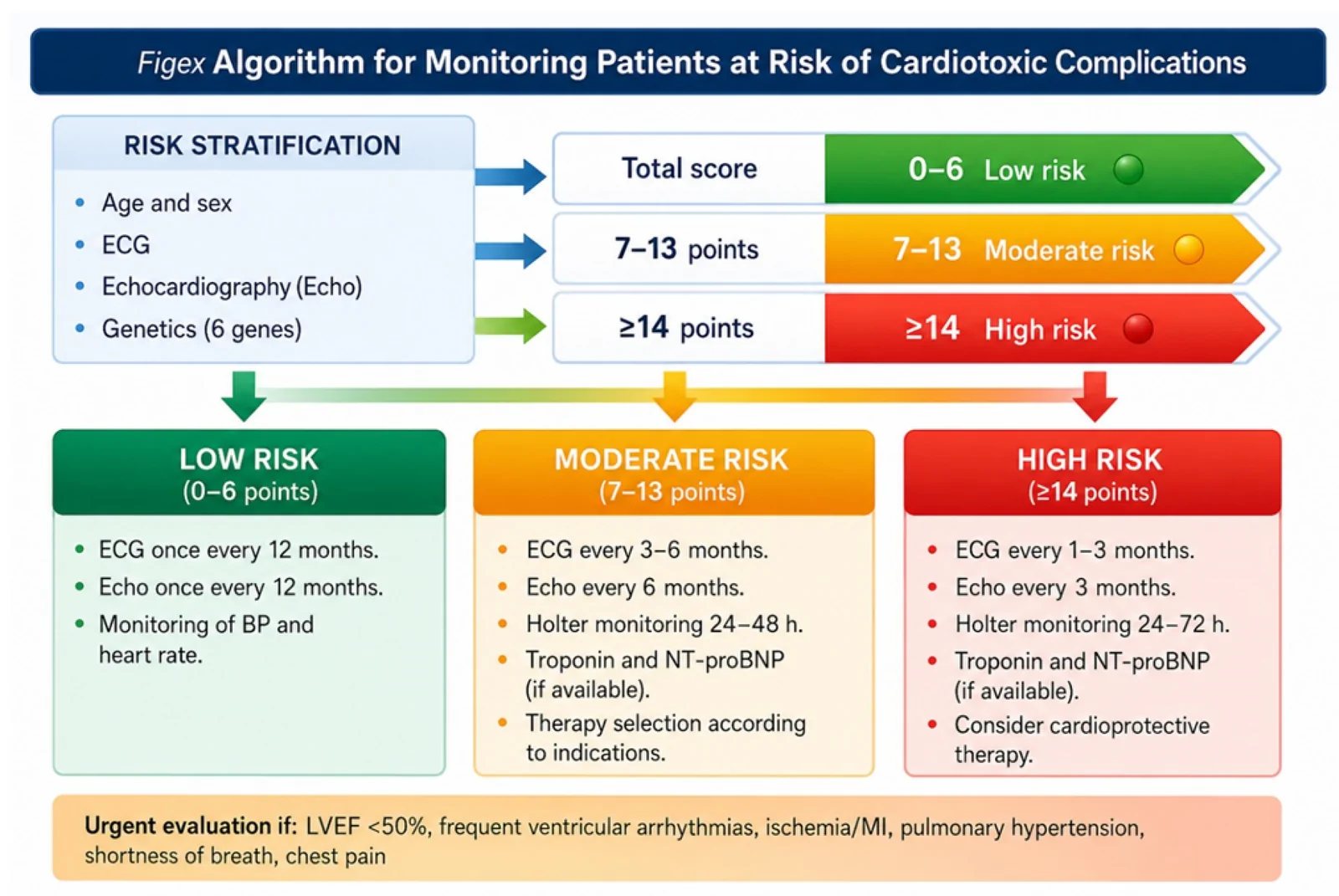
Figex algorithm for risk stratification and monitoring of patients at risk for cardiotoxic complications.

The proposed prognostic algorithm, incorporating age, sex, ECG, echocardiographic parameters, and cumulative genetic risk, may help identify patients at an increased risk of cardiotoxic complications and provide a rationale for personalized cardiac monitoring and early preventive strategies. However, the proposed combined genetic risk score and predictive algorithm should be regarded as exploratory and hypothesis-generating. Because the model was developed within a single study cohort and neither internal nor external validation was performed, its immediate clinical application cannot be recommended. Further validation in larger, independent, multicenter, prospective cohorts is required before implementation in routine clinical practice. These findings support the potential value of integrating clinical and genetic factors into future risk-stratification strategies for cardiotoxicity in patients with acute leukemia. The proposed risk-stratification and monitoring algorithm is presented in Figure 2.

## Figures and Tables

**Table 1:** Genetic polymorphisms remaining statistically significant after FDR correction.

Polymorphism	*p*
COMT Val158Met	<0.001
COL1A1 C1997A	0.001
SOD2 C14510A	0.010
EDN1 Lys198Asn	0.030

**Table 2:** Raw and FDR-adjusted *p*-values for the analyzed genetic polymorphisms.

Gene	Raw *p*	FDR-Adjusted *p*
COMT	<0.001	<0.001
COL1A1	0.001	0.004
SOD2	0.010	0.020
EDN1	0.030	0.045
GPX4	0.089	0.110
CAT	0.590	0.590

**Table 3:** Demographic and clinical characteristics of the patients.

Parameter	Value
Number of patients	89
Age, years	52 (36–63)
Female	53 (59.6 %)
Male	36 (40.4%)
Left ventricular hypertrophy (LVH)	52 (58.4%)
Reduced LVEF 50%	21 (23.6%)
Arrhythmias	41 (46.1%)
Ischemia/post-infarction cardiosclerosis	25 (28.1%)

**Table 4:** Association of age and sex with cardiac complications.

Factor	LVH (*p*-Value)	Arrhythmias (*p*-Value)	Reduced LVEF (*p*-Value)
Age	0.001	0.062	0.003
Sex	0.088	0.041	0.094

**Table 5:** Combined summary of allelic and genotypic distributions of GPX4, SOD2, COL1A1, CAT, EDN1, and COMT polymorphisms.

Gene	Variant	Patients, n (%)	Controls, n (%)	s	*p*	OR (95% CI)
GPX4 (C718T)	C	110 (53.9)	114 (58.8)	0.9	0.56	0.8 (0.55–1.22)
	T	94 (46.1)	80 (41.2)	0.9	0.56	1.2 (0.82–1.81)
	C/C	30 (29.4)	34 (35.1)	0.7	0.50	0.8 (0.43–1.40)
	C/T	50 (49.0)	46 (47.4)	0.1	0.90	1.1 (0.61–1.86)
	T/T	22 (21.6)	17 (17.5)	0.5	0.60	1.3 (0.64–2.62)
SOD2 (C14510A)	C	134 (65.7)	152 (78.4)	7.9	0.01	0.5 (0.34–0.82)
	A	70 (34.3)	42 (21.6)	7.9	0.01	1.9 (1.21–2.95)
	C/C	44 (43.1)	62 (63.9)	8.6	0.01	0.4 (0.24–0.75)
	C/A	46 (45.1)	28 (28.9)	5.6	0.08	2.0 (1.13–3.63)
	A/A	12 (11.8)	7 (7.2)	1.2	0.41	1.7 (0.65–4.51)
COL1A1 (C1997A)	C	131 (64.2)	159 (82.0)	15.8	0.01	0.4 (0.25–0.62)
	A	73 (35.8)	35 (18.0)	15.8	0.01	2.5 (1.60–4.00)
	C/C	43 (42.2)	66 (68.0)	13.4	0.01	0.3 (0.19–0.61)
	C/A	45 (44.1)	27 (27.8)	5.7	0.08	2.0 (1.14–3.68)
	A/A	14 (13.7)	4 (4.1)	5.6	0.08	3.7 (1.25–10.96)
CAT (G262A)	G	175 (85.8)	162 (83.5)	0.4	0.72	1.2 (0.69–2.06)
	A	29 (14.2)	32 (16.5)	0.4	0.72	0.8 (0.49–1.45)
	G/G	75 (73.5)	68 (70.1)	0.3	0.66	1.2 (0.64–2.20)
	G/A	25 (24.5)	26 (26.8)	0.1	0.86	0.9 (0.47–1.68)
	A/A	2 (2.0)	3 (3.1)	0.3	0.81	0.6 (0.10–3.78)
EDN1 (Lys198Asn)	Lys	151 (74.0)	159 (82.0)	3.6	0.21	0.6 (0.39–1.01)
	Asn	53 (26.0)	35 (18.0)	3.6	0.21	1.6 (0.99–2.58)
	Lys/Lys	56 (54.9)	64 (66.0)	2.5	0.34	0.6 (0.35–1.11)
	Lys/Asn	39 (38.2)	31 (32.0)	0.9	0.53	1.3 (0.74–2.36)
	Asn/Asn	7 (6.9)	2 (2.1)	2.7	0.35	3.5 (0.78–15.80)
COMT (Val158Met)	Val	104 (51.0)	85 (43.8)	2.0	0.29	1.3 (0.90–1.98)
	Met	100 (49.0)	109 (56.2)	2.0	0.29	0.7 (0.51–1.11)
	Val/Val	28 (27.5)	21 (21.6)	0.9	0.55	1.4 (0.72–2.62)
	Val/Met	48 (47.1)	43 (44.3)	0.1	0.72	1.1 (0.64–1.95)
	Met/Met	26 (25.5)	33 (34.0)	1.7	0.26	0.7 (0.36–1.22)

**Table 6:** Multivariable logistic regression analysis of genetic predictors of cardiotoxic complications.

Gene	Risk Allele/Genotype	β	OR	95% CI	*p*
COMT Val158Met	Met allele	1.34	3.83	2.11–6.95	0.001
COL1A1 C1997A	A allele	0.93	2.53	1.60–4.00	0.001
EDN1 Lys198Asn	Asn allele	0.58	1.78	1.05–3.01	0.030
SOD2 C14510A	A allele	0.64	1.90	1.21–2.95	0.010
GPX4 C718T	T allele	0.39	1.47	0.94–2.30	0.089
CAT G262A	A allele	0.05	1.05	0.58–1.89	0.590

**Table 7:** Model effectiveness.

Parameter	Value
Model $\chi^{2}$	31.8
Model *p*	<0.001
Nagelkerke $R^{2}$	0.42
AUC	0.85
Sensitivity	76.3%
Specificity	78.6%

**Table 8:** Association of individual polymorphisms with cardiac complications.

Gene (Variant)	Phenotype	OR	*p*-Value
GPX4 (T)	Reduced LVEF	2.3	0.021
SOD2 (A)	Arrhythmias	2.1	0.028
CAT (A)	Ischemia	2.0	0.037
COL1A1 (A)	Left ventricular hypertrophy (LVH)	2.6	0.009
EDN1 (Asn)	Pulmonary hypertension/tachycardia	2.2	0.031
COMT (Met/Met)	Arrhythmias	2.4	0.015

## Data Availability

The original contributions presented in this study are included in the article. Further inquiries can be directed to the corresponding author.
